# Supplementary material for: Non-pharmacological delirium detection and management interventions for informal caregivers of older people at home: A scoping review protocol
Source: PLoS One. 2024 Sep 20;19(9):e0308886. doi: 10.1371/journal.pone.0308886 (PMC11414944; doi:10.1371/journal.pone.0308886)
Supplement: S1 Appendix — (DOCX) [file pone.0308886.s001.docx]

### **S1 Appendix: Search strategy**

Database(s): Ovid MEDLINE(R) and In-Process, In-Data-Review & Other Non-Indexed Citations 1946 to June 04, 2024.
Search Strategy: run 2024-06-05.

| **#** | **Searches** | **Results** |
| --- | --- | --- |
| 1 | adult child*.mp. | 4635 |
| 2 | brother*.mp. | 15562 |
| 3 | caregiver*.mp. | 112802 |
| 4 | companion*.mp. | 21409 |
| 5 | daughter*.mp. | 29947 |
| 6 | famil*.mp. | 1548510 |
| 7 | friend*.mp. | 140793 |
| 8 | husband*.mp. | 43038 |
| 9 | (informal adj3 care*).mp. | 9256 |
| 10 | partner*.mp. | 234497 |
| 11 | peer*.mp. | 153459 |
| 12 | relative*.mp. | 1716640 |
| 13 | sibling*.mp. | 62965 |
| 14 | sister*.mp. | 46612 |
| 15 | son*.mp. | 215102 |
| 16 | spouse*.mp. | 35109 |
| 17 | volunteer*.mp. | 244527 |
| 18 | (wife or wives).mp. | 11467 |
| 19 | or/1-18 | 4145157 |
| 20 | deliri*.mp. | 25670 |
| 21 | confus*.mp. | 74167 |
| 22 | ((ICU or intensive care) adj3 psych*).mp. | 737 |
| 23 | or/20-22 | 97037 |
| 24 | clinic*.mp. | 6118682 |
| 25 | communit*.mp. | 862475 |
| 26 | health* centre*.mp. | 13218 |
| 27 | health* center*.mp. | 40423 |
| 28 | home*.mp. | 760079 |
| 29 | house*.mp. | 258828 |
| 30 | (independen* adj2 living).mp. | 17313 |
| 31 | primary care.mp. | 151689 |
| 32 | residence*.mp. | 102405 |
| 33 | ((transition or discharge*) adj3 (patient or home or communit*)).mp. | 76374 |
| 34 | or/24-33 | 7703575 |
| 35 | Family/ | 85945 |
| 36 | Adult Children/ | 1909 |
| 37 | Siblings/ | 13675 |
| 38 | Spouses/ | 11814 |
| 39 | Caregivers/ | 53111 |
| 40 | Friends/ | 7096 |
| 41 | Visitors to Patients/ | 2302 |
| 42 | Extended Family/ | 29 |
| 43 | Nuclear Family/ | 6045 |
| 44 | Peer Group/ | 24689 |
| 45 | or/35-44 | 190462 |
| 46 | Confusion/ | 5169 |
| 47 | Delirium/ | 12758 |
| 48 | Emergence Delirium/ | 846 |
| 49 | or/46-48 | 18239 |
| 50 | Primary Health Care/ | 94198 |
| 51 | Residence Characteristics/ | 38712 |
| 52 | Home Environment/ | 402 |
| 53 | Housing/ | 20572 |
| 54 | Housing for the Elderly/ | 1653 |
| 55 | Independent Living/ | 12400 |
| 56 | Community Health Centers/ | 7598 |
| 57 | Community Mental Health Centers/ | 3054 |
| 58 | community participation/ | 18652 |
| 59 | community-based participatory research/ | 5846 |
| 60 | community networks/ | 7214 |
| 61 | "delivery of health care"/ | 122885 |
| 62 | hospital to home transition/ or patient discharge/ | 41651 |
| 63 | or/50-62 | 356313 |
| 64 | 19 or 45 | 4146060 |
| 65 | 23 or 49 | 97037 |
| 66 | 34 or 63 | 7806745 |
| 67 | 64 and 65 and 66 | 7433 |
| 68 | exp Aged/ | 3508353 |
| 69 | Geriatrics/ | 31686 |
| 70 | 68 or 69 | 3526871 |
| 71 | elderly.tw,kw. | 301100 |
| 72 | geriatric*.tw,kw. | 80431 |
| 73 | senior.tw,kw. | 43920 |
| 74 | (older adj (adult? or m#n or wom#n or person? or people)).tw,kw. | 191483 |
| 75 | sexagenarian.tw,kw. | 75 |
| 76 | septuagenarian.tw,kw. | 245 |
| 77 | octogenarian.tw,kw. | 1851 |
| 78 | nonagenarian.tw,kw. | 744 |
| 79 | centenarian.tw,kw. | 916 |
| 80 | gerontolog*.tw,kw. | 9078 |
| 81 | or/71-80 | 559441 |
| 82 | 70 or 81 | 3692109 |
| 83 | 67 and 82 | 1991 |
| 84 | limit 83 to yr="1980 -Current" | 1963 |
