# Supplementary material for: Non-pharmacological delirium detection and management interventions for informal caregivers of older people at home: A scoping review protocol
Source: PLoS One. 2024 Sep 20;19(9):e0308886. doi: 10.1371/journal.pone.0308886 (PMC11414944; doi:10.1371/journal.pone.0308886)
Supplement: S2 Appendix — (DOCX) [file pone.0308886.s002.docx]

**S2 Appendix: Draft data extraction instrument**

Section 1: Authors, Year of Publication, country, language

Section 2: Type of source (theoretical / conceptual, empirical, clinical)

Section 3: Description of intervention

1. Name
2. Goal(s)
3. Study purpose
4. Target population and setting
5. Research design
6. Sample size and main characteristics
7. Components and activities
8. Materials used
9. Mode of intervention delivery
10. Dose of intervention delivery
11. Interventionist (who delivered intervention)
12. Outcomes reported
13. Outcome measurement tools
14. Study results
